# Survey on the current status of statistical cognition and teaching needs of Chinese medical students

**DOI:** 10.3389/fpubh.2025.1621667

**Published:** 2025-07-09

**Authors:** Hui Ouyang, Siyang Wang, Lingyun Huang

**Affiliations:** ^1^Department of Neurology, Peking University People’s Hospital, Beijing, China; ^2^Yanshou Community Health Service Center, Beijing, China; ^3^Yiyang Central Hospital, Yiyang, China

**Keywords:** statistics, medical students, statistics education, medical education, medical statistics

## Abstract

**Purpose:**

To assess medical students’ needs regarding statistics education and inform potential reforms in medical statistics teaching.

**Method:**

A self-administered questionnaire survey was conducted among 274 medical students from five Chinese institutions. The survey evaluated students’ attitudes toward statistics, their perceived mastery of statistical knowledge, and their perspectives on current teaching methods and desired improvements.

**Result:**

Main findings: (1) only 10.22% of students reported strong interest in statistics and 8.76% are hard-working, while 75.18% perceived it as difficult and 64.23% spend less than 3 h per week studying statistics. (2) Most statistics courses were delivered in large-class settings (70.07%) or via lecture-based learning (53.28%). The average practical course duration is 10.86 ± 12.85 h. For 42.34% of students, their schools or majors do not offer practical courses in statistics. (3) 83.21% reported difficulties in selecting the correct statistical methods and 68.61% of students use Excel for analysis. 71.53% of medical students expressed difficulties in interpreting statistical analysis results. Although most students can pass the statistic exam, but 34.31% have misused statistical methods in scientific research, and 28.47% affected by statistical misuse in scientific research. (4) A high proportion (88.32%) emphasized the need for statistical guidance in medical research projects and advocated for integrating clinical and research cases into coursework, 79.56% willing to accept blended learning and 78.1% considered case-based learning an effective teaching approach.

**Conclusion:**

Medical students are weak in both theory and practical statistical skills. Current teaching methods cannot stimulate students’ interest in learning statistics, and lack sufficient alignment with real-world applications due to excessive reliance on lecture-based instruction over case-based learning (CBL), necessitating reforms in medical statistics education.

## 1 Introduction

Statistics is a discipline that combines the principles and methods of probability theory and mathematical statistics, guiding medical students in research design, data collection, data analysis, and drawing conclusions. With the advent of information technology such as smart healthcare, big data, and artificial intelligence, it has become particularly important for medical students to choose reasonable and effective research methods. It can lay a necessary foundation for medical students to engage in medical research, write papers, and read literature (1–3). Medical students who are proficient in statistical methods can avoid errors and deviations in scientific research results during the process of data organization and analysis (4–6).

However, currently some medical students and researchers may not have ideal mastery of statistical methods. Studies have shown that inappropriate use of statistical methods is common in published studies, leading to irreproducibility and even misleading conclusions (7, 8). Therefore, there is an urgent need to strengthen training for medical students and researchers in data analysis and display (9). Improving the statistical level of medical students first requires attention to their statistical education. At present, the participation of students in teaching is low, and there are still problems such as low student acceptance and “statistical anxiety” in learning (10, 11). The traditional teaching method of statistics is relatively monotonous, emphasizing theory over practice, which limits students’ initiative and creativity in learning. Research has shown that (11), medical students prefer examples of using statistical methods around clinical practice rather than pure statistical theory teaching. The reform of the traditional teaching mode of statistics for medical students has become a consensus among some scholars (12–14).

Based on teaching practice and the schedules of some Chinese medical colleges, we found Chinese medical statistics education is typically compressed into a single preclinical semester (8–12 sessions total), most medical schools dedicating ≤30 contact hours to the subject. The curriculum emphasizes theoretical knowledge, while few programs include hands-on statistical software training. This pedagogical approach correlates with poor learning outcomes: a lot of medical students found statistics unengaging, and could not apply statistical methods to clinical practice (15). The early timing of instruction (predominantly Years 2–4) further exacerbates the theory-practice gap, as students lack clinical context during learning. There is an urgent need to integrate longitudinal, case-based statistical training.

Hence, there is an urgent need to understand the current situation of Chinese medical students’ mastery of statistics and propose methods for teaching reform in medical statistics. However, there is currently a lack of research in this area. Although a few studies have investigated the attitudes of medical students toward learning statistics (15, 16), the research content is relatively limited, mainly focusing on the attitudes and views of medical students toward statistics learning, without investigating the current situation of statistics education and the reform directions that students hope for. Among them, the representativeness of single center research is relatively limited (15). In addition, there is currently a lack of comprehensive research on the current situation of statistics learning among Chinese medical students. In years of medical teaching, the authors have also heard many medical students complain that the teaching method of medical statistics is difficult to understand, and even after completing the course, they still cannot apply statistical methods to solve clinical and scientific problems. However, few teachers care about their views on teaching medical statistics, and most teachers mainly focus on whether they can pass theoretical exams and earn credits.

Therefore, this article conducted a multicenter survey, to explore the current situation of statistical learning among medical students, to provide reference for promoting the reform of statistical teaching mode among medical students.

## 2 Materials and methods

### 2.1 Objects and study procedure

The participants of this study were medical students from five schools, including Peking University School of Medicine, Nanhua University, Tianjin Medical University, Xiangnan University and Changsha Medical College, from September to December 2023. Given the exploratory nature of this descriptive survey, a convenience sampling approach was adopted by recruiting intact class from 5 medical schools across China. Then we randomly selected 293 out of 1876 eligible students using SPSS 24.0 to ensure equal selection probability. Potential participants were contacted via email, phone calls, or in-person invitations based on their availability and institutional records. Only students who volunteered were enrolled in the study. Junior-year students who had not yet taken medical statistics courses were excluded. The survey utilized a validated self-designed scale with established reliability (*α* = 0.850) and construct validity (KMO = 0.74, I-CVI = 0.84, S-CVI = 0.92). Trained assistants distribute and collected questionnaires, they were available to explain the survey purpose and clarify any questions during the completion process. Questionnaires were completed through online or onsite at participants’ convenience. The sample size was determined using the formula for observation studies: *n* = (Zα/2)^2XP(1-P)/d^2, according to the results of pilot study, we required 245 participants, and our final sample exceeded this requirement. During the research process, 350 questionnaires were distributed and 293 valid questionnaires were collected. The questionnaire of non-medical related college students was excluded, incomplete questionnaires have been removed, and 274 questionnaires were retained. The survey was conducted anonymously.

A total of 274 medical students were included, with an average age of 23.76 ± 3.55 years old. Female students accounted for 56.93% and male students accounted for 43.07%. Undergraduate students in lower grades account for 5.84%, undergraduate students in higher grades account for 69.34%, master’s students account for 22.63%, and doctoral students account for 2.19%.

### 2.2 Statistic analysis

The data was analyzed using SPSS 24.0 statistical software. Descriptive statistical analysis was conducted on the questionnaire results. Quantitative data is expressed as mean ± standard deviation, while categorical variables are expressed as percentages. When comparing between groups, we used *t*-test or ANOVA methods.

## 3 Results

### 3.1 Subjective attitudes and understanding of statistics among medical students

**Figure 1:**
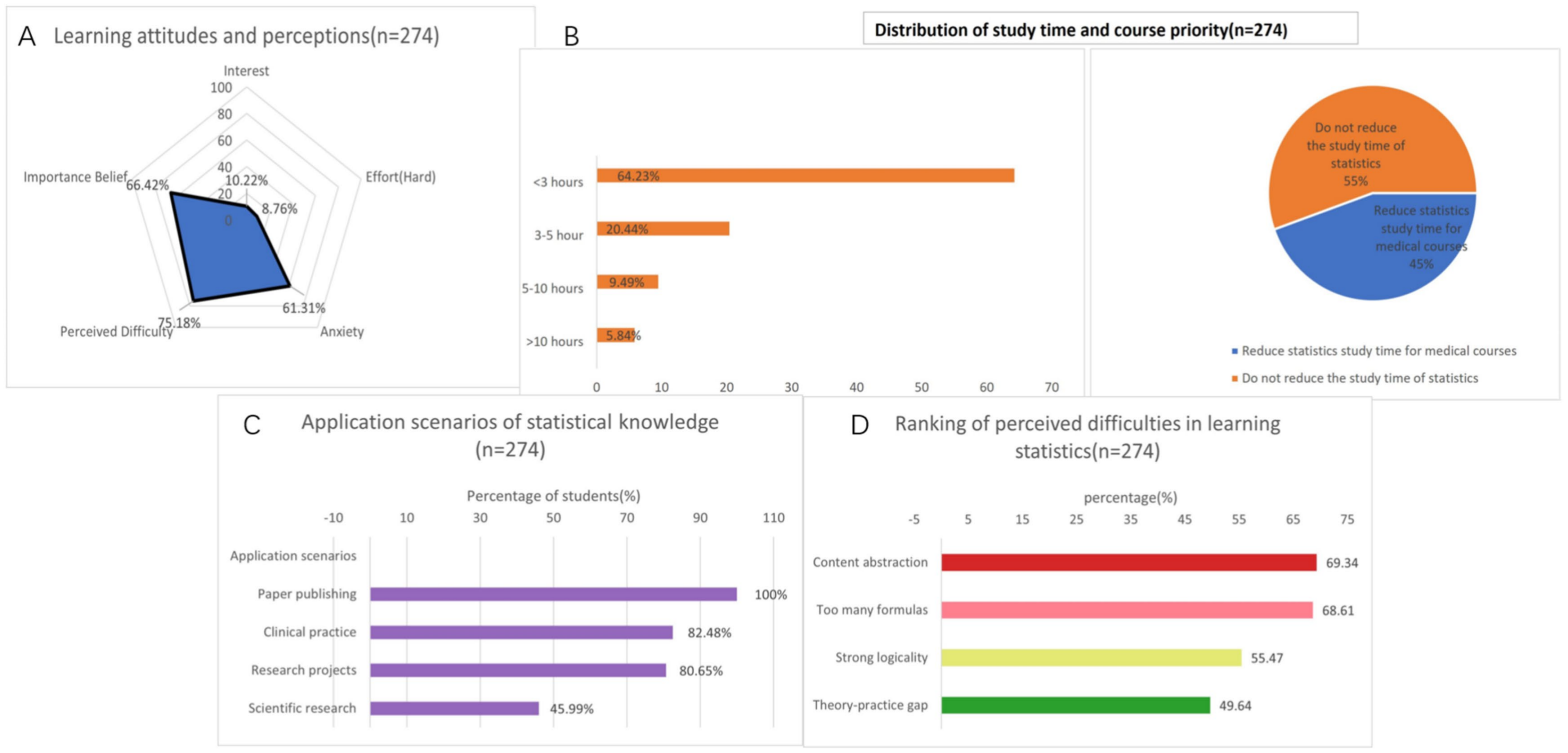
Integrated visualization of students’ subjective attitudes and understanding towards statistics. **(A)** Learning attitudes and perceptions. **(B)** Distribution of study time and course priority. **(C)** Application scenarios of statistical knowledge. **(D)** Ranking of perceived difficulties in learning statistics. *n* = 274.

According the results of the study, 10.22% of medical students expressed a strong interest in statistics. In terms of effort in learning statistics, 8.76% are very hard-working. 64.23% of medical students spend less than 3 h per week studying statistics. 61.31% of medical students feel anxious about learning statistics, 75.18% believe that learning statistics is difficult. In terms of understanding the importance of statistics, 66.42% believe that statistics are very important for medical students. 97.08% believe that statistics are helpful for the clinical and scientific research of medical students. 100% of students use statistics in their published papers, and 82.48% of students use statistical methods in clinical practice. In terms of the reasons for learning statistics and whether they are willing to take statistics as an elective, 70.8% of students are required to complete compulsory courses (Table 1 and Figure 1).

**Table 1:** General characteristics of survey subjects and their subjective attitudes and understanding toward statistics (*N* = 274).

Variables	Values
Age (y)	23.76 ± 3.55
Gender (male, %)	43.07
Major (clinical medicine, %)	80.29
Grade (%)	
Undergraduate lower grades	5.84
Undergraduate senior year	69.34
Postgraduate	22.63
Doctoral students	2.19
Interest in statistics (%)	10.22
Effort in learning statistics (%)	
Work hard	8.76
Moderate effort	71.53
Not hardworking	19.71
Reduce statistics study time in order to study medical courses (%)	44.55
Weekly study time for statistics (%)	
Over 10 h	5.84
5–10 h	9.49
3–5 h	20.44
Less than 3 h	64.23
Anxious about learning statistics (%)	61.31
Difficulty in learning statistics (%)	75.18
Believing that statistics are important for medical students (%)	66.42
Believing that statistics are helpful for medical students in clinical and scientific research (%)	97.08
The attitude toward learning statistics is influenced by the age, professional title, and major of the teaching staff (%)	
Yes	38.69
No	61.31
Willing to take statistics as an elective course (%)	40.15
The reasons for learning statistics (%)	
Completing compulsory courses	70.8
Scientific research needs	45.99
Using statistics in the project (%)	80.65
Using statistics in published papers (%)	100
Using statistical methods in clinical practice (%)	82.48
The reasons why medical students find it difficult to learn statistics (%)	
Too many formulas	68.61
Content abstraction	69.34
Strong logicality	55.47
The disconnect between theory and practice	49.64

### 3.2 The current situation of statistics education for medical students

**Figure 2:**
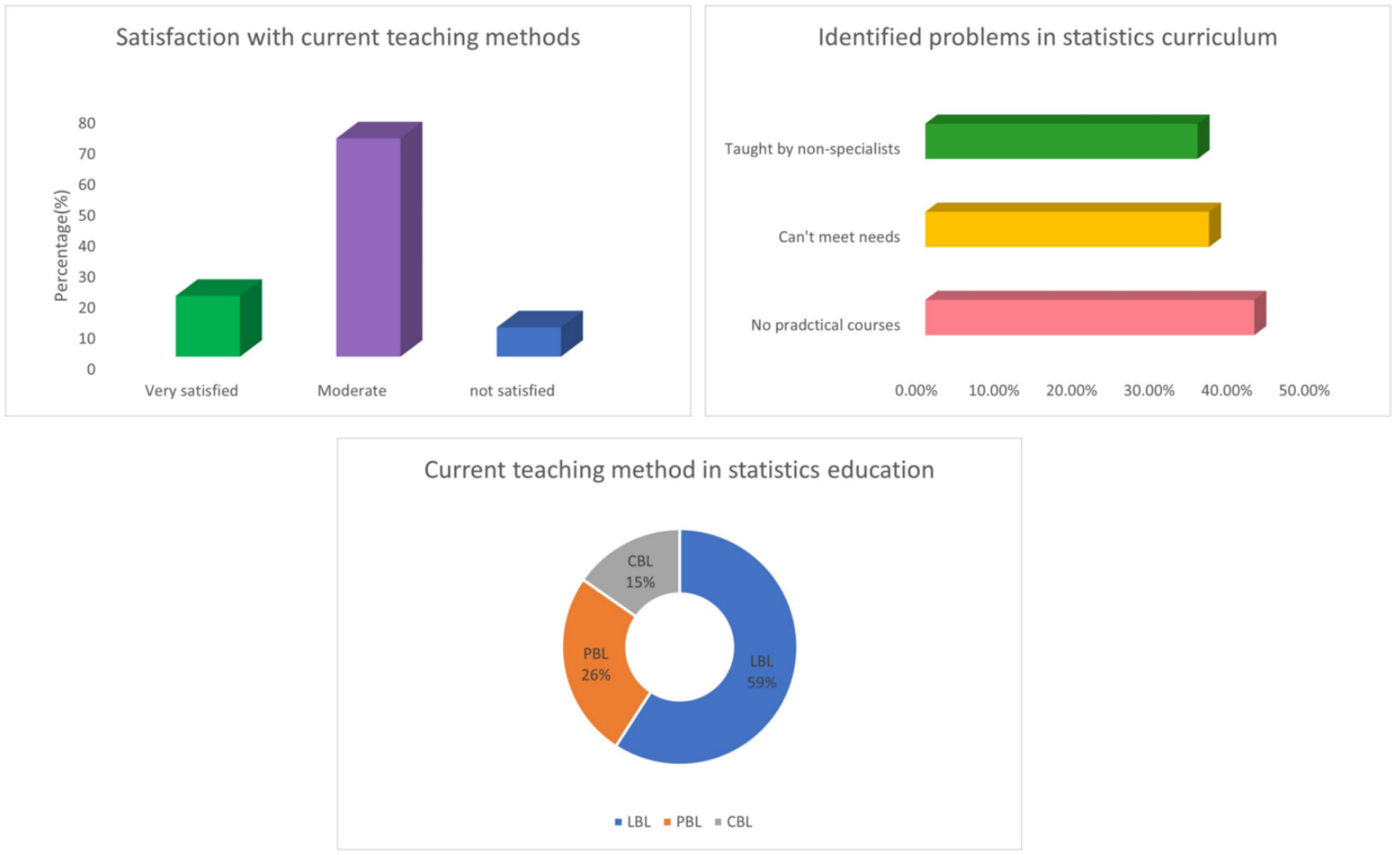
Integrated visualization of current status of statistics education for medical students (*n* = 274).

79.56% of medical students learn statistics through relevant school courses. In terms of learning statistical software, 71.53% of medical students learn through practical courses offered by the school. 70.07% of the statistics courses for medical students are taught in large classes. 59.12% of medical students in their schools use a lesson based approach (LBL) in statistics. The average practical course duration is 10.86 + 12.85 h. 48.91% of schools conduct statistical exams through theoretical exams. For 42.34% of students, their schools or majors do not offer practical courses in statistics. 58.39% of students mainly seek advice from non-statistical professionals when encountering problems (Table 2 and Figure 2).

**Table 2:** Current status of statistics education for medical students (*N* = 274).

Variable	Values
Ways to learn statistical design, analysis, and reporting standards (%)	
Related on campus courses (including on campus online courses)	79.56
Special lectures or training courses	34.31
Books or journal literature	37.96
Web page/forum/official account	30.66
Consult classmates/clinical physicians/mentors	42.34
Consult the statistics teacher	26.28
Ways to learn statistical software (%)	
Practical courses in schools	71.53
Self-study	47.45
Software Tutorials	30.66
Consult a teacher majoring in statistics	32.85
Consult non-statistics teachers	16.06
The statistics course is taught in large classes (%)	70.07
Adopting traditional classroom teaching (%)	53.28
The statistics classroom includes group learning/discussion (%)	58.39
The current methods used in school statistics teaching (%)	
Teaching method based on teaching (LBL)	59.12
problem-based learning (PBL)	25.55
Case-based teaching method (CBL)	15.33
The theoretical instructor is a non-statistical major (%)	26.28
The practical teaching teacher is a non-statistics major (%)	35.04
Statistics theory course hours (N ± SD)	26.81 ± 13.62
Statistics practice course hours (N ± SD)	10.86 ± 12.85
Methods of conducting statistical exams in schools (%)	
Theoretical Test	48.91
Practical assessment	13.87
Theoretical exam and practical assessment	37.23
Academic exchange opportunities are insufficient (%)	76.64
Satisfaction with current statistical teaching methods (%)	
Very satisfied	19.71
Moderate satisfaction	70.8
Not satisfied	9.49
Statistics is more difficult than other medical courses (%)	42.34
Statistics course cannot meet clinical or research needs (%)	36.5
Practical courses in statistics not offered (%)	42.34
Stage of receiving statistical education (%)	
Before engaging in clinical internships	60.91
During clinical internship	26.37
Resident physician	12.73
Have not received any statistical practice courses after clinical or scientific practice (%)	61.31
When encountering problems, seek help from statistical professionals (%)	40.88
Mainly seeking advice from non-statistical professionals (%)	58.39

### 3.3 The mastery of statistical knowledge by medical students

**Figure 3:**
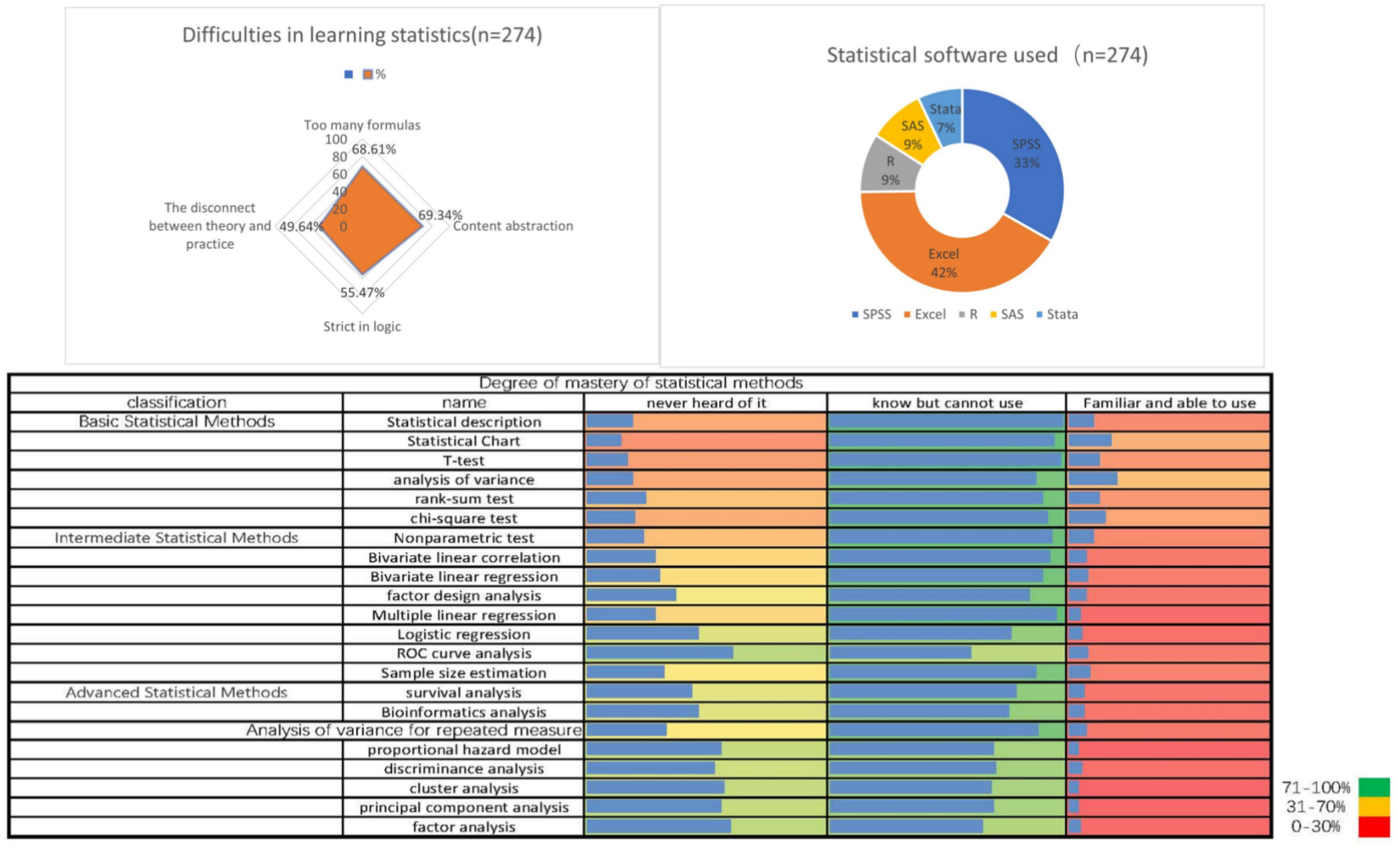
Integrated visualization of the current situation of students’ mastery of statistical knowledge (*n* = 274).

83.21% reported difficulties in selecting the correct statistical methods. In terms of the statistical software used, 68.61% of students will use Excel for analysis. When answering questions about difficulties in statistical application, 71.53% of medical students expressed difficulties in interpreting statistical analysis results professionally. 69.34% of students believe that statistics is abstract for medical students. 34.31% of students have misused statistical methods in scientific research, and 28.47% of students have been affected by statistical misuse in scientific research or graduation (Table 3 and Figure 3).

**Table 3:** The current situation of medical students’ mastery of statistical knowledge (*N* = 274).

Variable	Values
Difficulties in Scientific and Reasonable Statistical Design (%)	81.02
Difficulties in selecting the correct statistical methods (%)	83.21
Statistical software used	
SPSS	54.74
Excel	68.61
R	15.33
SAS	14.6
Stata	11.68
Difficulties in statistical applications (%)	
Professional interpretation of statistical analysis results	71.53
Scientific and reasonable statistical design	65.69
Correct selection of statistical methods in data analysis	53.28
Believing that medical students face difficulties in learning statistics (%)	
Too many formulas	68.61
Content abstraction	69.34
Strict in logic	55.47
The disconnect between theory and practice	49.64
Understanding of statistical knowledge (%)	
Statistical description	
Never heard of it	14.6
I know, but I do not know how to use it	75.91
Familiar and able to use	9.49
Statistical Chart	
Never heard of it	10.95
I know, but I do not know how to use it	72.99
Familiar and able to use	16.06
*p*-value	
Relatively familiar	24.09
General understanding	58.39
Not very familiar	17.52
*T*-test	
Never heard of it	13.14
I know, but I do not know how to use it	75.18
Familiar and able to use	11.68
Analysis of variance	
Never heard of it	14.6
I know, but I do not know how to use it	67.15
Familiar and able to use	18.25
Rank-sum test	
Never heard of it	18.98
I know, but I do not know how to use it	69.34
Familiar and able to use	11.68
Chi-square test	
Never heard of it	15.33
I know, but I do not know how to use it	70.8
Familiar and able to use	13.87
Non-parametric test	
Never heard of it	18.25
I know, but I do not know how to use it	72.26
Familiar and able to use	9.49
Bivariate linear correlation	
Never heard of it	21.9
I know, but I do not know how to use it	71.53
Familiar and able to use	6.57
Bivariate linear regression	
Never heard of it	23.36
I know, but I do not know how to use it	69.34
Familiar and able to use	7.3
Factor design analysis	
Never heard of it	28.47
I know, but I do not know how to use it	64.96
Familiar and able to use	6.57
Analysis of variance for repeated measurement design	
Never heard of it	25.55
I know, but I do not know how to use it	67.88
Familiar and able to use	6.57
Multiple linear regression	
Never heard of it	21.9
I know, but I do not know how to use it	73.72
Familiar and able to use	4.38
Survival analysis	
Never heard of it	33.58
I know, but I do not know how to use it	60.58
Familiar and able to use	5.84
Bioinformatics analysis	
Never heard of it	35.77
I know, but I do not know how to use it	58.39
Familiar and able to use	5.84
Proportional hazard model	
Never heard of it	43.07
I know, but I do not know how to use it	53.28
Familiar and able to use	3.65
Logistic regression	
Never heard of it	35.77
I know, but I do not know how to use it	59.12
Familiar and able to use	5.11
Discriminance analysis	
Never heard of it	40.88
I know, but I do not know how to use it	54.01
Familiar and able to use	5.11
Cluster analysis	
Never heard of it	43.8
I know, but I do not know how to use it	52.55
Familiar and able to use	3.65
Principal component analysis	
Never heard of it	43.07
I know, but I do not know how to use it	53.28
Familiar and able to use	3.65
ROC curve analysis	
Never heard of it	46.72
I know, but I do not know how to use it	45.99
Familiar and able to use	7.3
Factor analysis	
Never heard of it	45.99
I know, but I do not know how to use it	49.64
Familiar and able to use	4.38
Sample size estimation	
Never heard of it	24.82
I know, but I do not know how to use it	67.15
Familiar and able to use	8.03
Understanding statistical concepts	
Fully understand	5.84
Partial understanding	74.45
Not understand	19.71
Understanding statistical design	
Fully understand	9.49
Partial understanding	72.26
Not understand	18.25
Can clarify statistical analysis results in literature or research (%)	
Can	9.49
Partially possible	60.58
Cannot	29.93
Can evaluate the correctness of statistical analysis methods in literature or research (%)	
Can	10.22
Partially possible	57.66
Cannot	32.12
Misuse of statistical methods in scientific research (%)	34.31
Due to statistical misuse affecting scientific research or graduation (%)	28.47

### 3.4 Medical students’ expectations for statistical education and their demand for statistical learning

**Figure 4:**
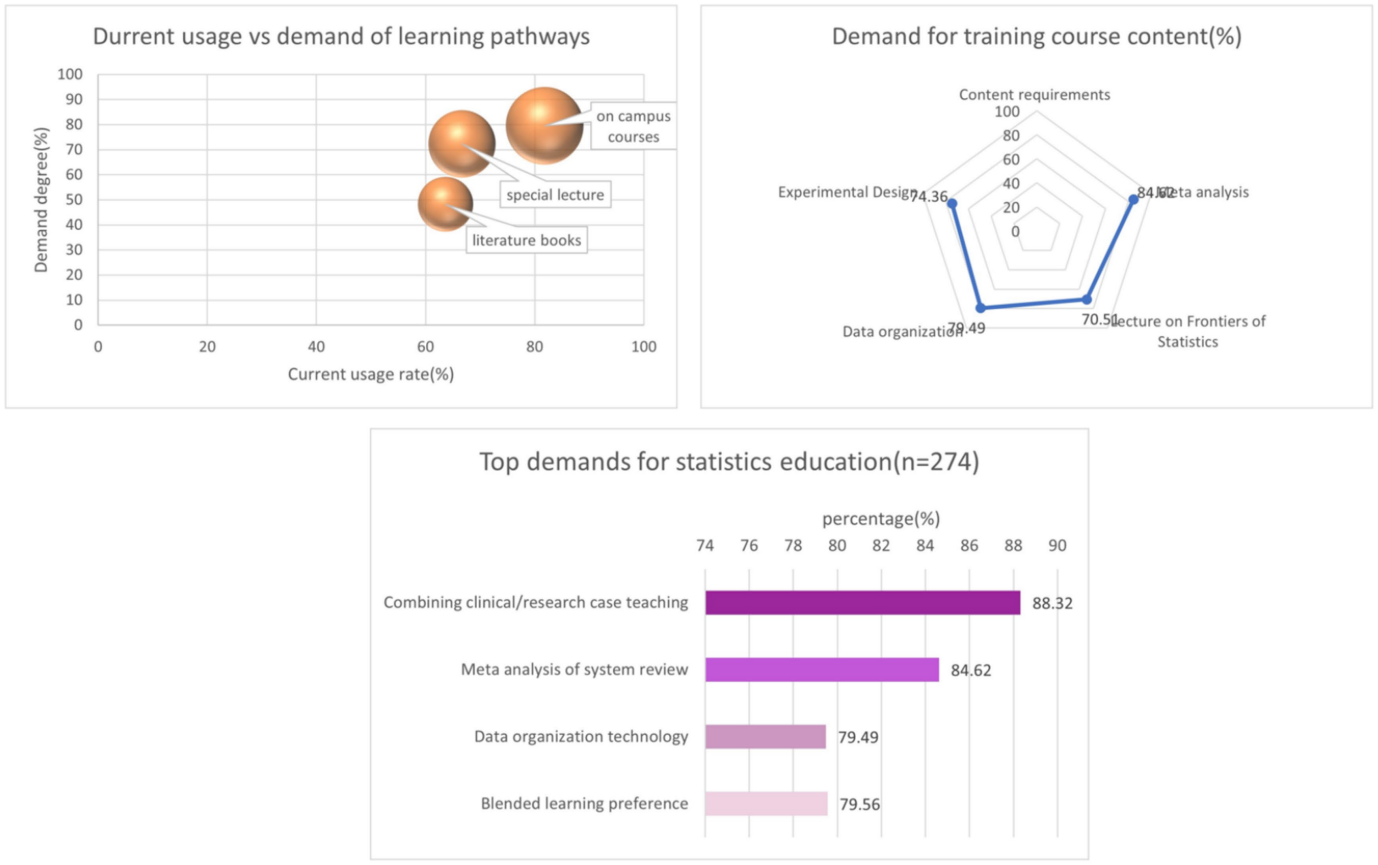
Integrated visualization of the demand of medical students for learning statistics (*n* = 274).

56.93% of students believe that short-term specialized statistical training courses should be offered. 88.32% of students believe that statistical guidance or consultation is needed in the development of the project, while 88.32% of medical students believe that the statistics course needs to incorporate clinical and research cases to combine with reality. In terms of preferred statistical teaching methods, 79.56% of students are willing to accept blended learning (Table 4 and Figure 4).

**Table 4:** The demand of medical students for learning statistics (*N* = 274).

Variable	Values
Short term specialized statistical training courses should be offered (%)	56.93
The content of the training course that hope to learn (%)	
Meta analysis of system evaluation	84.62
Lectures on the Frontiers of Statistical Development	70.51
Data collation	79.49
Experimental design	74.36
It is necessary to conduct statistical review before blind review of the thesis (%)	56.93
Statistical guidance or consultation is required during the project implementation (%)	72.26
The statistics course needs to incorporate clinical and research cases to integrate with reality (%)	88.32
The requirement for systematic learning of statistical analysis, design, and reporting standards (%)	48.18
Consider appropriate methods for teaching statistics (%)	
Case study	78.1
Classroom teaching	69.34
WeChat group interactive teaching	41.61
Network teaching	46.72
More willing to accept statistical teaching methods (%)	
Blended learning	79.56
Traditional classroom teaching	20.44
Ways to learn statistical design, analysis, and reporting standards (%)	
On campus courses (including online courses)	81.82
Special lectures or training courses	66.67
Books or journal literature	63.64
Web page or forum or official account	51.52
Seek advice from classmates, clinical physicians, or mentors	59.09
Consult the statistics teacher	50.00

### 3.5 Medical students’ gender differences in perceptions and competencies in statistics learning

Significant gender differences were revealed in statistics engagement among medical students (all *p* < 0.05). Female students reported higher clinical application of statistics (35.0% vs. 20.3% frequent use) and greater test proficiency (20.0% vs. 6.8% for chi-square), despite experiencing more difficulties in statistical design (86.3% vs. 74.6%). While 98.8% of females valued statistics for research versus 94.9% of males, more males attributed academic delays to statistical misuse (42.4% vs. 28.8%). Curriculum preferences differed markedly: 65.0% of females favored group learning (vs. 49.2%), whereas 47.5% of males advocated reducing statistics instruction time (vs. 28.8%). Assessment outcomes showed females excelled in combined exams (43.8% vs. 27.1%), while males predominated in theoretical testing (59.3% vs. 42.5%) (Table 5).

**Table 5:** Gender differences in perceptions and competencies in statistics (*N* = 274).

Variables	Male	Female	*p*-value
Reduce statistics study time in order to study medical courses (%)	47.46	28.75	<0.001
Believing that statistics are helpful for medical students in clinical and scientific research (%)	94.92	98.75	0.022
Willing to take statistics as an elective course (%)	44.07	38.75	0.032
Using statistical methods in clinical practice (%)			
Never	22.03	15.00	0.021
Sometimes	57.63	50.00	
Frequent	20.34	35.00	
The statistics classroom includes group learning/discussion (%)	49.15	65.00	0.01
The practical teaching teacher is a non-statistics major (%)	37.28	32.50	0.015
Methods of conducting statistical exams in schools (%)			
Theoretical test	59.32	42.50	0.011
Practical assessment	13.56	13.75	
Theoretical exam and practical assessment	27.12	43.75	
Statistics is more difficult than other medical courses (%)	37.29	47.50	0.003
Difficulties in Scientific and Reasonable Statistical Design (%)	74.58	86.25	0.019
Rank-sum test			
Never heard of it	16.95	21.25	0.003
I know, but I do not know how to use it	77.97	61.25	
Familiar and able to use	5.08	17.50	
Chi-square test			0.003
Never heard of it	13.56	17.50	
I know, but I do not know how to use it	79.66	62.50	
Familiar and able to use	6.78	20.00	
Due to statistical misuse affecting scientific research or graduation (%)	42.37	28.75	0.022
Short term specialized statistical training courses should be offered (%)	50.85	62.50	0.038
More class hours should be added	22.03	27.50	0.006

### 3.6 Disparities in statistical perceptions and competencies between undergraduate and postgraduate medical students

Postgraduates reported higher interest in statistics (25.71% vs. 5.77%, *p* < 0.001), greater belief in its importance for medical training (85.71% vs. 60.58%, *p* < 0.001), and more frequent use in research (91.31% vs. 55.54%, *p* < 0.001) and clinical practice (85.71% vs. 80.79%, *p* = 0.011). Despite exerting more effort (20.00% vs. 5.77% “work hard,” *p* < 0.001), postgraduates expressed higher anxiety (74.29% vs. 57.69%, *p* = 0.015). Competency gaps persisted: while postgraduates showed greater familiarity with advanced methods (e.g., logistic regression, ROC analysis, *p* < 0.001), both groups struggled to evaluate statistical methods in literature (≤11.54% “can” assess correctly). Demands for additional training (77.14% vs. 50.96%, *p* < 0.001) and dissatisfaction with teaching methods (≤22.12% “very satisfied”) highlighted unmet needs across cohorts (Table 6).

**Table 6:** Grade differences in perceptions and competencies in statistics (*N* = 274).

Variables	Undergraduate	Postgraduate	*p*-value
Interest in statistics (%)	5.77	25.71	<0.001
Effort in learning statistics (%)			
Work hard	5.77	20.00	<0.001
Moderate effort	71.15	71.43	
Not hardworking	23.08	8.57	0.021
Reduce statistics study time in order to study medical courses (%)	72.55	40.00	0.022
Anxious about learning statistics (%)	57.69	74.29	0.015
Believing that statistics are important for medical students (%)	60.58	85.71	<0.001
Believing that statistics are helpful for medical students in clinical and scientific research (%)	96.15	100.00	<0.001
The attitude toward learning statistics is influenced by the age, professional title, and major of the teaching staff (%)	34.62	54.29	0.005
Willing to take statistics as an elective course (%)	30.77	71.43	<0.001
Using statistics in the project (%)	55.54	91.31	<0.001
Using statistical methods in clinical practice (%)	80.79	85.71	0.011
Methods of conducting statistical exams in schools (%)			
Theoretical Test	53.85	37.14	0.011
Practical assessment	10.58	22.86	
Theoretical exam and practical assessment	35.58	40.00	
Statistics is more difficult than other medical courses (%)	66.67	57.14	0.001
Satisfaction with current statistical teaching methods (%)			
Very satisfied	22.12	14.29	0.044
Moderate satisfaction	70.19	68.57	
Not satisfied	7.69	17.14	
Practical courses in statistics not offered (%)	47.12	28.57	0.008
When encountering problems, seek help from statistical professionals (%)	38.46	51.43	0.003
*P*-value			
Relatively familiar	19.23	40.00	<0.001
General understanding	59.62	54.29	
Not very familiar	21.15	5.71	
*T*-test			
Never heard of it	13.46	14.29	<0.001
I know, but I do not know how to use it	78.85	60.00	
Familiar and able to use	7.69	25.71	
Rank-sum test			
Never heard of it	19.23	20.00	<0.001
I know, but I do not know how to use it	73.08	54.29	
Familiar and able to use	7.69	25.71	
Chi-square test			
Never heard of it	17.31	11.43	0.049
I know, but I do not know how to use it	71.15	65.71	
Familiar and able to use	11.54	14.39	0.011
Bivariate linear correlation			
Never heard of it	25.96	11.43	
I know, but I do not know how to use it	69.23	77.14	
Familiar and able to use	4.81	11.43	
Bivariate linear regression			
Never heard of it	27.88	11.43	0.010
I know, but I do not know how to use it	66.35	77.14	
Familiar and able to use	5.77	11.43	
Multiple linear regression			
Never heard of it	25.00	14.29	0.033
I know, but I do not know how to use it	72.12	77.14	
Familiar and able to use	2.88	8.57	
Survival analysis			
Never heard of it	39.42	20.00	0.011
I know, but I do not know how to use it	54.81	74.29	
Familiar and able to use	5.77	5.71	
Bioinformatics analysis			
Never heard of it	41.35	22.86	0.005
I know, but I do not know how to use it	51.92	74.29	
Familiar and able to use	6.73	2.86	
Proportional hazard model			
Never heard of it	47.12	34.29	0.012
I know, but I do not know how to use it	50.96	52.52	
Familiar and able to use	1.92	3.60	
Logistic regression			
Never heard of it	44.23	11.43	<0.001
I know, but I do not know how to use it	51.92	80.00	
Familiar and able to use	3.85	5.04	
Cluster analysis			
Never heard of it	50.96	25.71	0.001
I know, but I do not know how to use it	46.15	68.57	
Familiar and able to use	2.88	5.71	
Principal component analysis			
Never heard of it	48.08	31.43	0.041
I know, but I do not know how to use it	49.04	62.86	
Familiar and able to use	2.88	5.71	
ROC curve analysis			
Never heard of it	53.85	25.71	<0.001
I know, but I do not know how to use it	42.31	54.29	
Familiar and able to use	3.81	20.00	
Factor analysis			
Never heard of it	51.92	31.43	<0.001
I know, but I do not know how to use it	46.15	57.14	
Familiar and able to use	1.92	11.43	
Can evaluate the correctness of statistical analysis methods in literature or research (%)			
Can	11.54	8.57	0.015
Partially possible	51.92	71.43	
Cannot	36.54	20.00	
Misuse of statistical methods in scientific research (%)	30.77	48.57	0.009
term specialized statistical training courses should be offered (%)	50.96	77.14	<0.001
Statistical guidance or consultation is required during the project implementation (%)	67.31	88.57	<0.001
The requirement for systematic learning of statistical analysis, design, and reporting standards (%)	42.31	68.57	<0.001

### 3.7 Disparities in statistics education and research application between tiered medical universities

Students from key universities demonstrated higher utilization of statistics in research projects (85.19% vs. 60.02%, *p* < 0.001) and greater familiarity with advanced statistical methods, including *t*-tests (17.44% vs. 3.37% “familiar and able to use,” *p* = 0.001) and ANOVA (23.84% vs. 10.38%, *p* = 0.006). Key universities reported more frequent statistical guidance during project implementation (78.49% vs. 63.21%, *p* = 0.008) and stronger demand for systematic statistics training (54.07% vs. 40.57%, *p* = 0.036). Teaching environments differed significantly, with non-key universities more likely to employ non-specialist instructors (practical courses: 38.68% vs. 31.98%, *p* = 0.034) and use large-class teaching (79.25% vs. 65.12%, *p* = 0.015). Both groups showed limited practical competency, though key university students exhibited better understanding of multivariate techniques (principal component analysis: 4.65% vs. 1.89% “familiar,” *p* = 0.002) (Table 7).

**Table 7:** Statistics engagement and teaching differences between key and non-key universities.

Variables	Key university	Non-key university	*p*-value
Using statistics in the project (%)	85.19	60.02	<0.001
The attitude toward learning statistics is influenced by the age, professional title, and major of the teaching staff (%)	45.35	30.19	0.016
Believing that statistics are important for medical students (%)	68.02	65.09	0.044
The statistics course is taught in large classes (%)	65.12	79.25	0.015
The statistics classroom includes group learning/discussion (%)	52.91	66.98	0.024
Statistics is more difficult than other medical courses (%)	44.19	41.51	0.034
The theoretical instructor is a non-statistical major (%)	21.52	33.01	0.021
The practical teaching teacher is a non-statistics major (%)	31.98	38.68	0.034
Practical courses in statistics not offered (%)	34.88	39.62	0.008
*T*-test			
Never heard of it	10.47	18.87	0.001
I know, but I do not know how to use it	72.09	77.36	
Familiar and able to use	17.44	3.37	
Analysis of variance			
Never heard of it	11.63	20.75	0.006
I know, but I do not know how to use it	64.53	68.87	
Familiar and able to use	23.84	10.38	
Rank-sum test			
Never heard of it	17.44	22.64	0.027
I know, but I do not know how to use it	66.28	71.70	
Familiar and able to use	16.28	5.66	
Not satisfied	7.69	17.14	
Practical courses in statistics not offered (%)	47.12	28.57	0.008
When encountering problems, seek help from statistical professionals (%)	38.46	51.43	0.003
*P*-value			
Relatively familiar	19.23	40.00	<0.001
General understanding	59.62	54.29	
Not very familiar	21.15	5.71	
*T*-test			
Never heard of it	13.46	14.29	<0.001
I know, but I do not know how to use it	78.85	60.00	
Familiar and able to use	7.69	25.71	
Rank-sum test			
Never heard of it	19.23	20.00	<0.001
I know, but I do not know how to use it	73.08	54.29	
Familiar and able to use	7.69	25.71	
Bivariate linear regression			
Never heard of it	18.60	32.08	0.015
I know, but I do not know how to use it	72.09	64.15	
Familiar and able to use	9.30	3.77	
Bioinformatics analysis			
Never heard of it	40.70	30.19	0.044
I know, but I do not know how to use it	55.81	60.38	
Familiar and able to use	3.49	9.43	
Proportional hazard model			
Never heard of it	52.33	30.19	<0.001
I know, but I do not know how to use it	43.02	67.92	
Familiar and able to use	4.65	1.89	
Principal component analysis			
Never heard of it	51.16	32.08	0.002
I know, but I do not know how to use it	44.19	66.04	
Familiar and able to use	4.65	1.89	
Statistical guidance or consultation is required during the project implementation (%)	78.49	63.21	0.008
The requirement for systematic learning of statistical analysis, design, and reporting standards (%)	54.07	40.57	0.036

## 4 Discussion

### 4.1 Main findings: theory-practice gap in medical students’ statistical education

This study reveals significant gaps in medical statistics education, with 83.21% of students struggling to select appropriate statistical methods and 71.53% facing interpretation challenges, leading to widespread misuse (34.31% in research projects). Didactic teaching dominates (70.07% large-class lectures; 59.12% LBL), while 42.34% of programs lack practical training, exacerbating disengagement (only 10.22% show strong interest). Assessment focuses excessively on theoretical exams (48.91%), failing to evaluate applied competencies. Despite recognizing statistics’ importance (97.08%), students report anxiety (61.31%) and inadequate preparation for research needs. Comparative analyses highlight global alignment in these challenges, though this study provides the most comprehensive multi-center evidence. Critically, 88.32% demand clinically-integrated case teaching, while 79.56% prefer blended learning. These findings underscore an urgent need for curriculum reform prioritizing active learning, longitudinal software training, and competency-based assessment. The study revealed significant gender disparities, with female students demonstrating greater statistical engagement (35.00% frequent clinical use vs. 20.34% males) and method familiarity (17.50% proficient in rank-sum test vs. 5.08% males), while postgraduate students showed substantially higher competency than undergraduates (91.31% vs. 55.54% project usage). Institutional analysis identified resource disparities, with key universities exhibiting better faculty qualifications (21.52% vs. 33.01% non-statistics instructors) and research integration (85.19% vs. 60.02% project engagement), highlighting systemic inequalities in statistical education.

### 4.2 The problems and needs of statistical learning for medical students

#### 4.2.1 Poor knowledge and practical ability in statistical theory

According to the results of the study, 83.21% reported difficulties in selecting the correct statistical methods. 68.61% of students still use Excel for analysis. 71.53% of medical students expressed difficulties in interpreting statistical analysis results professionally. 34.31% of students have misused statistical methods in scientific research. From the results, it can be concluded that the current statistical theoretical knowledge and practical abilities of medical students are not ideal. In the context of big data analysis and the development of information science, medical students mainly focus on basic statistical analysis methods in their understanding of statistical theory knowledge, lacking understanding of commonly used statistical methods in current medical research. This reflects that the statistical theory knowledge learned by medical students no longer meets the current needs of medical research, and the statistical theories taught by schools have not been adjusted accordingly according to the development of medicine, falling behind the times. In addition, medical students mostly stay at the level of “having heard of but not knowing how to use” statistical concepts and methods, and most of the statistical software they use is basic statistical software with incomplete functions such as Excel and SPSS. This reflects that the statistical theoretical knowledge mastered by medical students has not been translated into practical abilities. A considerable number of medical students have misused statistical methods, which may even affect their research projects, paper publications, and graduation blind reviews. The above results indicate that the statistical theory and practical abilities of medical students are insufficient. It is urgent to improve the statistical theory and practical abilities of medical students (9).

#### 4.2.2 Lack of initiative and interest in statistical learning

According the results of the study, only 10.22% of medical students expressed a strong interest in statistic, 64.23% of medical students spend less than 3 h per week studying statistics. 61.31% of medical students feel anxious about learning statistics. 97.08% believe that statistics are helpful for the clinical and scientific research of medical students. The research results show that the majority of medical students believe that learning statistics is important and that learning statistics is helpful for their clinical and scientific research. However, medical students generally lack interest in statistics, and only a small proportion of them have a strong interest in statistics. Medical students find it difficult to learn statistics and feel anxious and fearful about it (17). At the same time, although medical students recognize the importance of statistics, most medical students are unwilling to take statistics as an elective and spend less time on statistics learning in their daily lives. Students are the main body of learning and teaching activities. All external factors such as teacher led teaching content and methods need to be influenced by internal factors. Milic et al. (11) and Kiekkas et al. (18) found lack of interest in learning can affect the level of understanding of statistics among clinical students. The level of interest and familiarity of clinical students with hygiene courses is related (16). Therefore, unfamiliarity with statistics and difficulty in mastering it may in turn reduce the interest of medical students in learning statistics.

#### 4.2.3 Statistics teaching basically meets the learning conditions, but there are still shortcomings

According the survey, 79.56% of medical students learn statistics through relevant school courses. 70.07% of the statistics courses for medical students are taught in large classes. 59.12% of medical students in their schools use a lesson based approach (LBL) in statistics. The average practical course duration is 10.86 ± 12.85 h. For 42.34% of students, their schools or majors do not offer practical courses in statistics. 58.39% of students mainly seek advice from non-statistical professionals when encountering problems. To conclude, it was found that the curriculum of medical schools provides the most basic theoretical and methodological support for statistical learning. However, there are still shortcomings. In a few cases, non-statistics teachers will provide statistical teaching to students, indicating that there is a lack of sufficient statistics professional teachers to participate in the teaching of statistical theory and practice to medical students under current conditions. Most statistics courses are taught using traditional classroom teaching and lecture based teaching methods (LBL). Under this teaching mode, the subjectivity of students is not valued, and it is not conducive to cultivating their interest in learning statistics (19, 20). Statistics courses have strong logic and systematicity, requiring a lot of time to explain basic concepts, applicable materials and application conditions of various research methods. Insufficient class hours may lead to insufficient understanding of statistical knowledge by students, which may be one of the reasons why medical students think statistics is difficult. Compared with theoretical courses, practical statistics courses are allocated fewer hours, which deprives students of the opportunity to think about the research design, data analysis, and organization of practical medical research problems, choose the correct research methods, and provide answers. The arrangement of statistics teaching for medical students is mostly before they are exposed to clinical practice, resulting in artificial isolation between the time of receiving statistics teaching and clinical and research practice.

#### 4.2.4 The assessment method is not comprehensive

The survey results show that the statistical assessment methods for medical students are mainly based on their final exam scores, 48.91% of schools conduct statistical exams through theoretical exams. The examination format is mostly a theoretical exam, which rarely testing their understanding of statistical research methods and problem-solving abilities in practical research. The examination method of statistics for medical students is too single, which on the one hand promotes students to pay too much attention to basic concepts in mastering the course, and on the other hand, there is less examination of whether students can flexibly grasp statistical knowledge and use it in practice, which is not conducive to cultivating students’ ability to solve clinical and scientific research practical problems in future learning and work.

#### 4.2.5 The demand of medical students for statistical education

56.93% of students believe that short-term specialized statistical training courses should be offered, while 88.32% of medical students believe that the statistics course needs to incorporate clinical and research cases to combine with reality. 79.56% of students are willing to accept blended learning. According to the results of this survey, most medical students have recognized the importance of statistics and have a willingness to learn statistics. Contrary to the current situation of medical statistics that emphasizes theory over practice and is disconnected from practice, most medical students hope to adopt a blended learning approach in statistics teaching, believing that case teaching is the most suitable method for statistics teaching. Medical students generally attach great importance to the practical application of statistics. Most medical students hope to offer statistical lectures or training courses to learn topics closely related to medical research. This indicates that medical students have realized that there is a certain gap between receiving current statistical education and proficiently applying statistical methods to clinical and scientific research practice. At the same time, medical students hope to obtain professional statistical evaluation before blind review of their papers and during the development of their research projects, which suggests that lax statistical review of research may be one of the important factors restricting the development of statistical learning.

### 4.3 Strategies and suggestions for statistical education for medical students

#### 4.3.1 Steadily promoting the construction of statistical courses for medical majors and building a good statistical learning platform

High-quality textbooks are the foundation of high-quality teaching. In addition, attention should be paid to the development of statistics and statistical software internship supporting textbooks. Firstly, it is necessary to adjust the content of statistics courses, reduce obscure and difficult to understand statistical principles, and increase more practical content, such as statistics in scientific research design and practical operation of statistical software. The second is to fully utilize the Internet and university resources, continuously provide free statistical software download channels for medical students, and establish a network platform for statistical software learning and practice. Teachers adopt a blended learning approach to guide, inspire, and supervise students, while encouraging them to be proactive and creative in the learning process (21, 22). The third is to change the implementation method of the curriculum, emphasizing the combination of online and offline teaching methods, organically combining classroom teaching with network platforms. The fourth is to introduce policies to encourage relevant enterprises to develop highly specialized and cost-effective statistical learning products. The fifth is to build a medical statistics case library that includes clinical practice cases and published research papers, for medical students to refer to. The sixth is to regularly organize medical statistics special lectures and statistical academic exchange meetings that meet the actual research needs of medical students.

#### 4.3.2 Guiding statistical talents to teach in medical schools and departments, creating favorable conditions for medical students to learn statistics

At present, only a small portion of statistics professionals enter medical colleges, resulting in a relative shortage of statistics professionals in medical colleges. On the one hand, this is related to the career choices of statistical professionals, and on the other hand, it is also related to the insufficient attention paid by medical colleges to statistical teachers and statistical education. Therefore, it is necessary to strengthen relevant publicity to make medical colleges aware of the necessity of admitting teachers majoring in statistics to teach. In the teaching of statistical theory and practice, it can be attempted to use the method of synchronous teaching and mutual supplementation by teachers with statistical expertise and clinical research experience, striving to achieve accurate statistical methods while combining medical clinical and research practical problems, helping students to have a deeper understanding of the statistical analysis methods they have learned.

#### 4.3.3 Teachers strengthen educational guidance to improve the efficiency and quality of statistical learning for medical students

Medical college teachers should change their ideological concepts and attach importance to statistical teaching. In statistics teaching, by incorporating practical clinical and medical research cases PBL and CBL teaching methods, students can cultivate their scientific research thinking based on clinical problems, and cultivate interest in statistical learning through self-learning and discussion. Increasing the proportion of statistical software hands-on operations in the course and encouraging students to actively participate in software operations during experimental classes can improve learning efficiency and enhance their enthusiasm for learning. Students should be personally involved in the experimental design, data collection, data organization and analysis, and paper writing process of specific research projects in reality. The experience gained from scientific research practice will help to understand the content of classroom teaching and provide a foundation for students’ future medical research. At the same time, guide and follow up on their use and feedback, so that students can develop good habits of self-directed learning. Improve the application ability of statistical software in clinical and scientific research. Finally, statistics teachers should participate in the project design, data analysis, and paper writing of medical students.

#### 4.3.4 Classroom teaching, teaching different content according to the different characteristics and learning requirements of students

According to the research results, the statistical level of medical students varies, and there are certain differences in their understanding of medical statistics. Some graduate students hope to conduct short-term training courses in statistics to learn advanced statistical analysis methods in a targeted manner to meet their research needs. The main purpose of some students studying statistics is to understand basic statistical knowledge and obtain graduation certificates through exams. Therefore, it is recommended to divide statistics teaching into basic and advanced classes for separate instruction.

#### 4.3.5 Improve assessment methods and create a diversified teaching evaluation system based on output oriented effectiveness benchmark education philosophy

Create a diversified teaching evaluation system to establish a multi-dimensional, comprehensive and systematic evaluation method around course objectives, and set assessment methods based on the different requirements of initial, intermediate, and higher-order objectives. For basic knowledge, multiple choice questions, right or wrong questions, fill in the blank questions, short answer questions, and other methods are often used for evaluation; for comprehensive application problems that require systematic and in-depth thinking, methods such as case analysis and scenario analysis are often used for evaluation; For software practical skills, they are often evaluated through hands-on operation.

### 4.4 Heterogeneity-driven reforms for statistical education in medical training

The observed gender and academic-level disparities in statistical competencies likely stem from multifaceted factors: women’s greater statistical engagement may reflect evolving medical education dynamics that increasingly reward collaborative learning styles, while undergraduates’ limited research exposure explains their poorer performance compared to postgraduates – a gap exacerbated by traditional curricula delaying research training until advanced years. The institutional resource disparities between key and non-key universities mirror global patterns of unequal STEM investment, particularly problematic for statistics education requiring specialized faculty and computational infrastructure. These findings mandate three policy interventions: (1) gender-sensitive pedagogy reforms emphasizing clinical applications to bridge male students’ engagement gap, (2) longitudinal research curricula starting in undergraduate years to build gradual statistical proficiency, and (3) national standardization of statistical education resources to mitigate institutional inequities, potentially through shared digital platforms and faculty exchange programs in medical schools. The alarmingly high statistical misuse rates across all subgroups further underscore the urgent need for competency-based assessment frameworks replacing current exam-focused evaluation systems.

### 4.5 Comparison with similar studies in other countries

A study conducted among medical students of Serbia (23) indicated that implementation of blended learning approaches can be considered as and attractive, cost-effective, and efficient alternative to classroom training in medical statistics, which is consistent with the results of our study. However, the research in Serbia only compared the impact of blended learning versus traditional classroom teaching in medical students’ statistical exam scores, focusing solely on test results without addressing their practical statistical skills. MacDougall et al. (24) conducted a survey among England medical student, mainly focuses on the importance of statistics. The majority of the surveyed medical students considered learning statistics to be very important, which is consistent with the findings of this study. However, this study did not investigate the existing problems in statistical education for medical students or potential directions for improvement. A study conducted in Iran (25) attempted to incorporate game-based methods into statistics education for medical students and explored the impact of this approach on their learning outcomes. This finding indirectly suggests that traditional statistics teaching methods may have inherent limitations. The few studies conducted in other countries generally align with the findings of the present study. However, compared to our research, these researches have relatively narrower scopes, primarily focusing on students’ perceptions of statistics or specific teaching methods, lacking a comprehensive investigation into the current status and potential improvements in statistical education for medical students. Moreover, most of them were single-center investigations.

### 4.6 Contribution to the national statistical teaching policies and strategies for reforming medical statistics education

This study identifies critical gaps in medical statistics education and proposes a comprehensive reform strategy to align training with modern research needs. Our findings reveal four key areas for policy intervention: (1) curriculum modernization through integration of advanced methods with foundational theory and modular curricula (basic→advanced→research-specific); (2) pedagogical reform via blended learning (23), clinical-integrated training during rotations, and case-based active learning to address student disengagement; (3) resource development by training specialized instructor teams (26), providing open-access platforms with statistical tools/case libraries, and incentivizing EdTech partnerships for medical-statistics simulations; and (4) assessment restructuring through multimodal evaluations (research projects, peer reviews, competency badges) and national standards for hands-on training. To implement these reforms, we recommend a phased approach: pilot testing in select medical schools, faculty certification programs, and policy dialogs with accreditation bodies. These evidence-based strategies – addressing curriculum-practice gaps, pedagogical inefficiencies, and resource limitations – can transform statistical education into a cornerstone of rigorous medical research, ultimately improving research validity and clinical decision-making. The proposed reforms balance immediate feasibility (e.g., using existing clinical cases for teaching) with long-term systemic change (e.g., national competency standards), offering a actionable roadmap for policymakers to elevate statistical proficiency across medical education.

### 4.7 Strength and limitation of the study

This research employed a multicenter, questionnaire-based design to comprehensively assess statistical education across China’s geographic and economic regions, enhancing the generalizability of findings. While anonymous surveys and rigorous interviewer training mitigated potential response biases. The research use questionnaire surveys to collect data, which may susceptible to response bias, including social desirability bias, acquiescence bias, courtesy bias and fear bias, which may compromise the validity and reliability of the findings. In order to mitigate these biases, we have taken the following measures: (1) the questionnaire survey was conducted anonymously. (2) A pilot study was conducted before the formal investigation to ensure that students could accurately understand the questionnaire. (3) Unified training was provided to all interviewers. Despite these limitations, the questionnaire surveys remain valuable for capturing subjective experiences and attitudes of medical students to statistics education. The percentage of undergraduate lower grades and doctoral students were relatively low, the sample size may not big enough. However, the study selected students randomly, and the selected samples were representative and roughly consistent with the proportion of students in each grade level according to the national data report.

## 5 Conclusion

Our study documents systemic shortcomings in medical statistics education, Predominant lecture-based teaching and inadequate practical training fail to meet students’ expressed needs for case-based and blended learning. These findings mandate: (1) replacing theoretical exams with competency assessments, (2) integrating statistical software training with clinical datasets, and (3) adopting active learning pedagogies. The study provides actionable evidence for curriculum reforms to bridge the theory-practice gap, ultimately enhancing research rigor and healthcare evidence quality.

## Data Availability

The raw data supporting the conclusions of this article will be made available by the authors, without undue reservation.
